# Klippel–Trénaunay syndrome with profound abdominal lymphatic-venous malformation in a three-day-old newborn: a case report and literature review

**DOI:** 10.3389/fped.2024.1326909

**Published:** 2024-01-19

**Authors:** Shih Yang Wei, Yu Peng Liu, Dao Chen Lin, Pei Shan Tsai

**Affiliations:** ^1^ Department of Radiology, MacKay Memorial Hospital, Taipei City, Taiwan; ^2^ Department of Radiology, Mackay Memorial Hospital, Hsinchu Branch, Hsinchu City, Taiwan; ^3^ Department of Radiology, Taipei Veterans General Hospital, Taipei City, Taiwan; ^4^ Division of Endocrine and Metabolism, Department of Medicine, Taipei Veterans General Hospital, Taipei City, Taiwan; ^5^ School of Medicine, National Yang Ming Chiao Tung University, Taipei City, Taiwan; ^6^ Department of Medicine, Mackay Medical College, New Taipei City, Taiwan; ^7^ Mackay Junior College of Medicine, Nursing and Management, New Taipei City, Taiwan

**Keywords:** Klippel–Trénaunay syndrome, newborn, lateral marginal vein, vein of Servelle, lymphatic-venous malformation

## Abstract

**Background:**

Klippel–Trénaunay syndrome, a kind of congenital limb-length-discrepancy disorder, is commonly associated with a variety of vascular anomalies.

**Case presentation:**

We present the case of a three-day-old newborn with a profound abdominal mass lesion during prenatal magnetic resonance imaging (MRI) examination. After delivery, physical examination revealed mild hemihypertrophy of the left lower extremity and red lesions on the left thigh. MRI of the abdomen showed a cyst-like lesion measuring 6.3 cm × 2.7 cm × 5.5 cm in the upper abdomen. Within the mass, there were also some possible calcified spots exhibiting high T1WI signals and low T2WI signals. A computed tomography (CT) scan of the abdomen was consistent with an ill-defined cystic tumor with small calcifications and encasement of mesenteric vessels. A MRI of the left lower extremity showed a tubular structure with a signal void and homogeneous strong enhancement located in the anterior subcutis of the left lower limb. The CT scan confirmed that the tubular structure was consistent with a venous malformation. This patient had features of Klippel–Trénaunay syndrome, including port-wine stains, a profound abdominal mass, and vascular malformations of the left lower extremity.

**Conclusions:**

In this article, we presented a case of Klippel–Trénaunay syndrome, emphasizing both prenatal and confirmatory postnatal cross-sectional imaging findings. The rare presentation of an abdominal lymphatic-venous formation played a pivotal role as a crucial indicator for an early diagnosis of Klippel–Trénaunay syndrome.

## 1 Introduction

Klippel–Trénaunay syndrome (KTS) is a rare congenital disorder that is characterized by the triad of venous malformations, cutaneous capillary malformations, lymphatic malformation and limb overgrowth (1). The incidence of KTS is very low and is estimated to be about 1:100,000 (1). The disease has no apparent ethnic or sex predilection (2, 3). We present a newborn case of KTS exhibiting the classical features and a rare profound abdominal mass.

## 2 Case description

**Figure 1:**
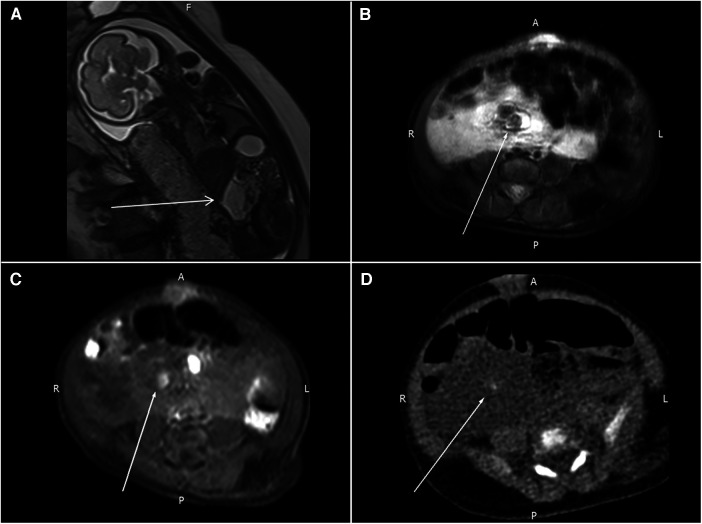
(**A**) Prenatal MRI shows a mass lesion with a high T2WI signal in the right abdomen (arrow) compressing the adjacent small bowel loops. (**B,C**) Follow-up MRI reveals a nodular lesion (arrows) with hypointensity in the T2WI sequence and hyperintensity in the T1WI sequence. (**D**) One tiny, calcified spot (arrow) in the pre-contrast CT images.

**Figure 2:**
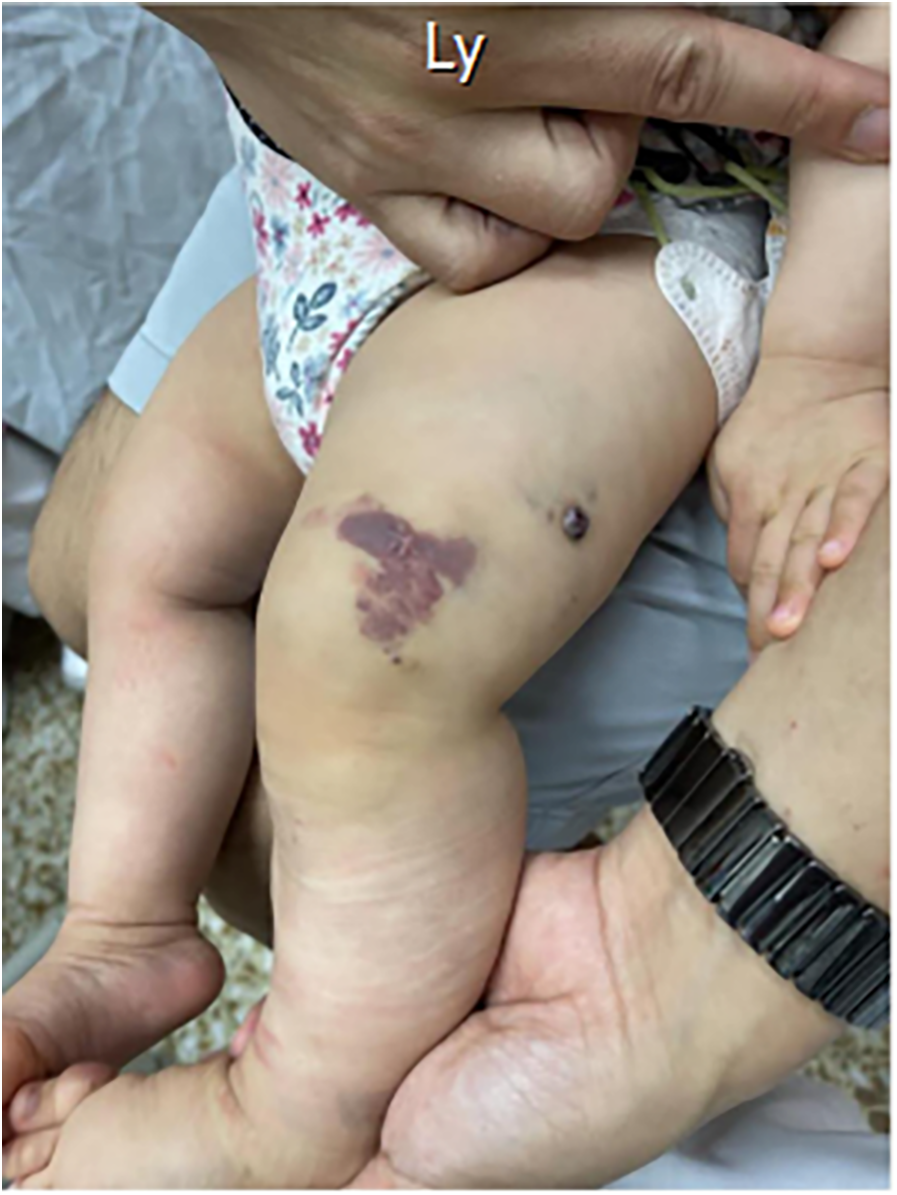
The port-wine stains (capillary malformations) on the left thigh.

A three-day-old girl presented with an abdominal mass lesion that was noted during routine prenatal ultrasound screening. At 26 weeks, the prenatal magnetic resonance imaging (MRI) was performed and showed a defined mass lesion in the right abdomen compressing the adjacent small bowel loops (Figure 1A). She was born at 39 weeks via vaginal delivery and weighed 3,142 g. There was no family or parental history of inherited vascular disorders. Physical examination revealed mild hemihypertrophy of the left lower extremity and red lesions on the left thigh (Figure 2).

A follow-up MRI of the abdomen was obtained after delivery, and this showed a 6.3 cm × 2.7 cm × 5.5 cm cyst-like lesion in the upper abdomen. Some possible calcified spots with high T1WI signals and low T2WI signals were also noted within the mass (Figures 1B,C). A computed tomography (CT) scan of the abdomen was consistent with an ill-defined cystic mass with small calcifications and encasement of mesenteric vessels. Lymphatic-venous malformation (LVM) was strongly suspected (Figure 1D).

**Figure 3:**
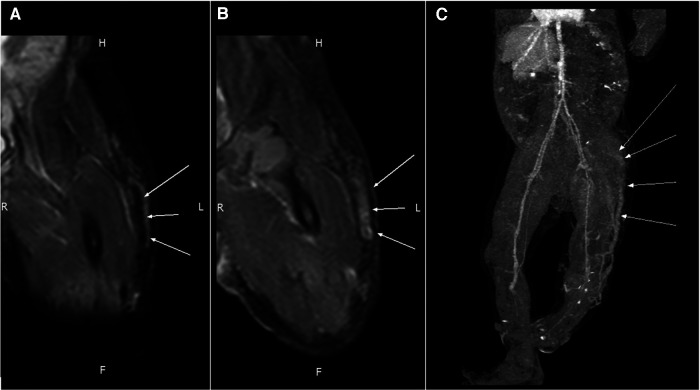
An MRI of the tubular structure at the anterior subcutis of the left thigh reveals (**A**) a signal void (arrows) in PDWI and (**B**) homogeneous strong enhancement (arrows) in the T1WI + C sequence. (**C**) Contrast-enhanced CT shows an engorged tubular structure (arrows) on the lateral aspect of the left lower limb that extends from the lower leg to the gluteal and pelvic regions.

An MRI of the left lower extremity revealed a tubular structure at the anterior subcutis of the left lower leg that extended to the lateral thigh subcutis and into the ipsilateral gluteus muscle, connecting with vessels at the left pelvic sidewall. The tubular lesion displayed a signal void and homogeneous strong enhancement, compatible with a vascular malformation (Figures 3A,B).

**Figure 4:**
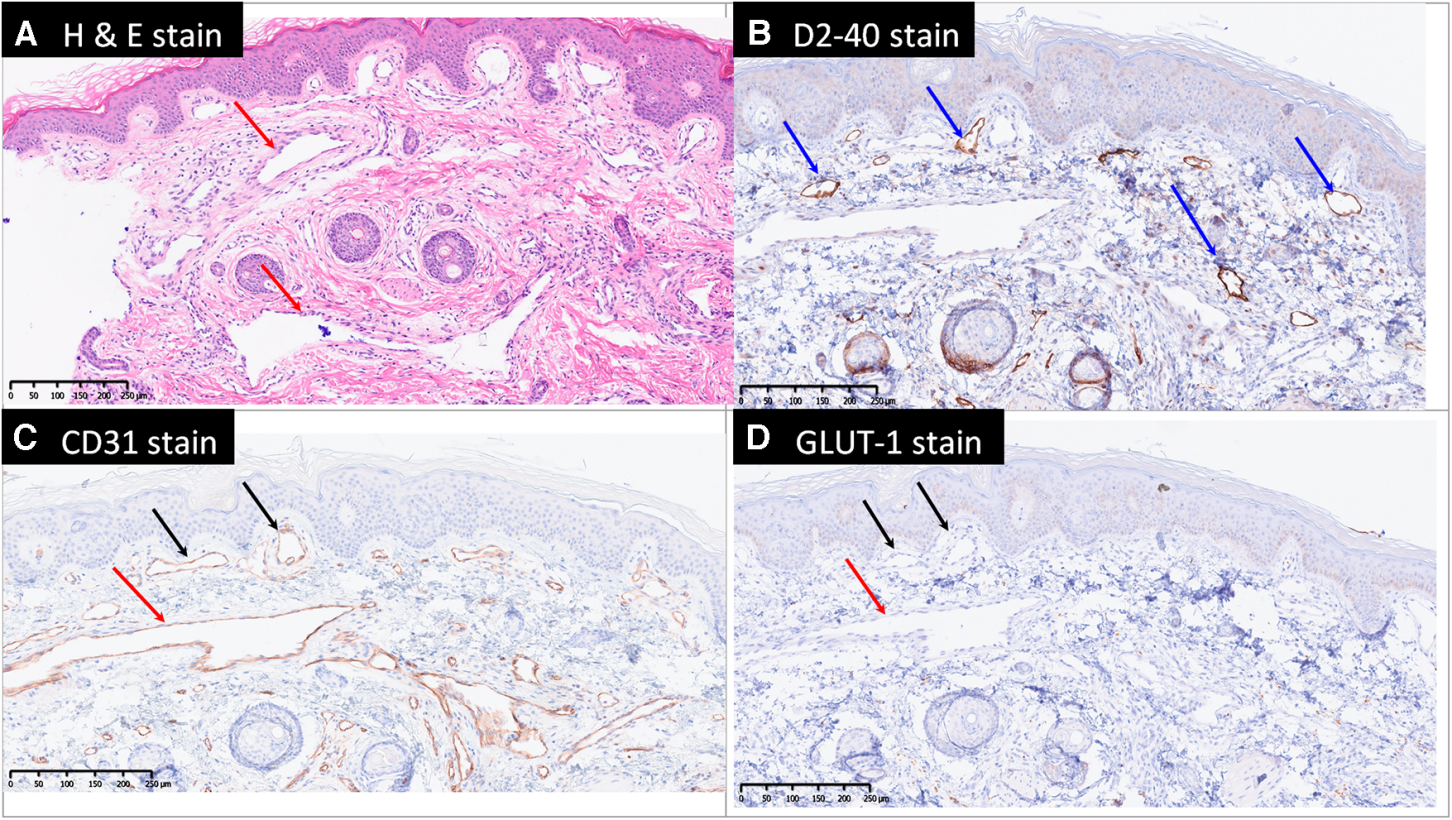
Microscopically, there are dilated capillaries (black arrows), lymphatics (blue arrows) and venules (red arrows) in the papillary dermis, reticular dermis and subcutis, shown by hematoxylin and eosin stain (H & E stain, **A**), D2-40 (**B**) and CD31 (**C**) stains. The blood vessels are negative for Glut1 (**D**) therefore exclude the diagnosis of infantile hemangioma. GLUT-1, Endothelial glucose transporter 1.

A CT scan of the left lower extremity also revealed a vascular structure with no significant enhancement in the arterial phase but gradual contrast filling in the delayed phase, indicating venous rather than arteriovenous malformation. Reconstructed images revealed one engorged incompetent vessel on the lateral aspect of the left lower extremity that extended from the lower leg to the gluteal and pelvic regions. This vessel is known as the lateral marginal vein or vein of Servelle (Figure 3C). Subcutaneous soft tissue stranding and edematous change from the left thigh to calf were also noted. A biopsy of the left thigh lesion was performed and the pathology revealed capillary–lymphatic–venous malformation (CLVM) (Figure 4). The findings of above were consistent with a diagnosis of KTS.

At 29 days old, the patient was therefore referred to the cardiovascular surgeon and the pediatric hematologist for treatment consultation. Initially, she only received oral beta-2 antagonist and compression therapy of lower extremity as management for KTS after discussing with patient's family. She regularly underwent follow-up at cardiovascular surgeon and the pediatric hematologist departments every three months, but no obvious improvement found during the follow up period. At 10 months old, she started to receive Sirolimus simultaneously. After several sessions of combination treatment (beta-2 antagonist, Sirolimus and compression therapy), regression of the left lower extremity edema was observed. The abdominal mass lesion exhibited a slight reduction in size during the ultrasound follow-up conducted after three months. At the time of writing, the patient was 18 months old and in regular follow-up at these out-patient departments every three months.

## 3 Discussion

Klippel–Trénaunay syndrome (KTS) is a rare congenital capillary–lymphatic–venous condition characterized by the following clinical triad: capillary malformations (port-wine stains), congenital venous or veno-lymphatic malformations, and bone and/or soft-tissue hypertrophy (4). A phenotypic diagnosis of KTS requires the presence of only two of these three cardinal features (1). The radiographic appearance of soft tissue, bone, and vessels can be assessed using plain films, sonograms, or CT and MRI (5, 6).

The capillary malformation in KTS is usually pink or purple in color, which is often described as a port-wine malformation. The venous or veno-lymphatic malformation is extensive and does not involve high-flow arteriovenous malformation (AVM). Liguori et al. (2) reported that 70% of KTS patients have a “vein of Servelle,” also known as a lateral marginal vein, extending from the foot or ankle to the infra-inguinal region. Both malformations typical of KTS were present in our case, who had port-wine stain as well as the lateral marginal vein in the left lower extremity.

In genetic studies, KTS is associated with the PI3K/AKT/mTOR signaling pathway and Parkes Weber syndrome (PWS) is a defect in the RASA-1 gene (1). The two syndromes have many features in common, such as cutaneous port-wine stains, asymmetrically enlarged limbs, and vascular malformations, which can lead to misdiagnosis. The critical clinical difference between these two disorders is the type of vascular malformation. PWS is associated with high-flow AVMs whereas KTS is associated with low-flow (venous and/or lymphatic) malformations (1). Images of the present case revealed venous malformation but not AVM, further confirming the diagnosis of KTS.

Kocaman et al. (7) reported that splenic hemangioma (venous malformation) may occur in KTS patients, and is often asymptomatic. However, in the present case, the profound mass lesion was located in the mesenteric region rather than the spleen. Further, the tumor was initially detected in prenatal screening and was suspected to be LVM. This early manifestation of capillary–lymphatic–venous abnormality may be another key to diagnosing KTS.

In 1900, the French physicians Klippel and Trénaunay classified KTS into four levels of severity (3). In accordance with the traditional classification, our case was categorized as class I (venous/phlebectasic dysplasias).

According to Sikakulya et al. (8), KTS can be associated with several complications, such as deep venous thrombosis, bleeding, pulmonary embolism, stasis dermatitis, cellulitis, and limb enlargement. Although no definitive treatment for KTS has been approved (9), an early approach consisting of multidisciplinary management should be considered. Macrocystic lymphatic malformations can be treated with coil embolization or sclerotherapy, while microcystic malformation might be treated only with oral sirolimus or doxycycline (1, 2). In addition, laser therapy is used to reduce the port-wine stains. All these management approaches are primarily symptomatic treatment and prevent further complications (10). Prognosis depends on the severity of the malformations and associated anomalies.

## 4 Conclusion

We report a case of KTS in a newborn with a profound abdominal LVM. She presented with the classical KTS features of capillary and venous malformations, which allowed her condition to be distinguished from PWS. Early distinction between KTS and PWS was possible based on the identification of significant hemodynamic arteriovenous fistulas, which is essential for further treatment and prognosis prediction. Additionally, she presented with a unique abdominal LVM in prenatal MRI, which served as an important clue for early diagnosis of KTS. The prognosis of KTS is variable and needs to be treated with multidisciplinary management.

## Data Availability

The original contributions presented in the study are included in the article/Supplementary Material, further inquiries can be directed to the corresponding author.
